# Differential Proteomics for Distinguishing Ischemic Stroke from Controls: a Pilot Study of the SpecTRA Project

**DOI:** 10.1007/s12975-018-0609-z

**Published:** 2018-01-24

**Authors:** A. M. Penn, V. Saly, A. Trivedi, M. L. Lesperance, K. Votova, A. M. Jackson, N.S. Croteau, R. F. Balshaw, M. B. Bibok, D. S. Smith, K. K. Lam, J. Morrison, L. Lu, S. B. Coutts, C. H. Borchers

**Affiliations:** 1 Department of Neuroscience, Stroke Rapid Assessment Unit (SRAU), Island Health, 1 Hospital Way, Victoria, BC V8Z 6R5 Canada; 2 Department of Mathematics and Statistics, University of Victoria, 3800 Finnerty Rd., Victoria, BC V8P 5C2 Canada; 3 Department of Research and Capacity Building, Island Health, 1952 Bay Street, Victoria, BC V8R 1J8 Canada; 4 Division of Medical Sciences, University of Victoria, Victoria, BC V8W 2Y2 Canada; 5 University of Victoria-Genome British Columbia Proteomics Centre, Vancouver Island Technology Park, #3101-4464 Markham St., Victoria, BC V8Z 7X8 Canada; 6 PROOF Centre of Excellence, 166-1081 Burrard Street, Vancouver, BC V6Z 1Y6 Canada; 7 BC Centre for Disease Control, 655 West 12th Avenue, Vancouver, BC V5Z 4R4 Canada; 8 Departments of Clinical Neurosciences, Radiology, and Community Health Services, Hotchkiss Brain Institute, Foothills Medical Centre, University of Calgary, 1403 - 29 Street N.W., Calgary, AB T2N 2T9 Canada; 9 Department of Biochemistry and Microbiology, University of Victoria, Petch Building Room 207, 3800 Finnerty Rd., Victoria, BC V8P 5C2 Canada

**Keywords:** Proteomics, Plasma proteins, Mass spectrometry, Stroke, Hematologic tests, Infarction

## Abstract

A diagnostic blood test for stroke is desirable but will likely require multiple proteins rather than a single “troponin.” Validating large protein panels requires large patient numbers. Mass spectrometry (MS) is a cost-effective tool for this task. We compared differences in the abundance of 147 protein markers to distinguish 20 acute cerebrovascular syndrome (ACVS) patients who presented to the Emergency Department of one urban hospital within < 24 h from onset) and from 20 control patients who were enrolled via an outpatient neurology clinic. We targeted proteins from the stroke literature plus cardiovascular markers previously studied in our lab. One hundred forty-one proteins were quantified using MS, 8 were quantified using antibody protein enrichment with MS, and 32 were measured using ELISA, with some proteins measured by multiple techniques. Thirty proteins (4 by ELISA and 26 by the MS techniques) were differentially abundant between mimic and stroke after adjusting for age in robust regression analyses (FDR < 0.20). A logistic regression model using the first two principal components of the proteins significantly improved discrimination between strokes and controls compared to a model based on age alone (*p* < 0.001, cross-validated AUC 0.93 vs. 0.78). Significant proteins included markers of inflammation (47%), coagulation (40%), atrial fibrillation (7%), neurovascular unit injury (3%), and other (3%). These results suggest the potential value of plasma proteins as biomarkers for ACVS diagnosis and the role of plasma-based MS in this area.

## Introduction

In the management of acute cerebrovascular syndrome (ACVS) [1], the high prevalence of conditions that mimic stroke presents a challenge, particularly for first-line physicians. Such mimics include migraine, Todd’s paresis following seizure, delirium, compressive neuropathies, and many other entities [2]. Unlike cardiology where an ECG and single blood test allows effective filtering, the first step with ACVS may be advanced imaging and/or specialist referral.

The development and validation of a reliable blood biomarker test capable of distinguishing ACVS from mimic has been challenging, despite numerous multi-center studies of varying size [3–11]. Additionally, most stroke biomarker studies have used ELISA technology, with the exception of some studies using newer methods for protein quantification such as mass spectrometry (MS) [12, 13]. ELISA uses immunoassay to measure protein expression but each protein requires a separate assay, even when a few are bundled together in a composite test. In contrast, MS allows simultaneous quantitation of large numbers of biomarkers, in a rapid, reproducible and sensitive assay at low cost per sample; the drawback being that low-abundance proteins remain a challenge to detect using MS. To date, no protein biomarkers have been successfully adopted into clinical practice, although commercial ELISA kits for stroke do exist, as performance has not been adequate [14].

In this paper, we report on a small-scale exploratory case-control study to examine the natural abundance and variability of 147 candidate plasma proteins in ischemic stroke and stroke-mimic patients. These candidate proteins include markers of stroke and cardiovascular disease selected through a comprehensive literature review of stroke biomarker research published prior to 2014 [15, 16]. We were required by our funders to examine published markers as discovery research is not eligible for this type of translational research funding. The objectives of this small-scale case-control study are (1) to confirm that the MS proteomic platforms yield useful information and (2) provide a preliminary vetting of many of the candidates for our eventual protein biomarker panel in development for transient ischemic attack (TIA) or mild stroke. This work is part of a larger SpecTRA study [17, 18], a large-scale, multi-site, precision medicine project that uses MS to measure 141 proteins concurrently in a clinical research program involving 1860 patients, to verify and validate a clinically useful blood test for TIA and minor stroke. As well, we chose to study severe stroke in this small-scale study on the premise that this would provide a more robust target than TIA, in terms of greater differential abundance of up- or down-regulated proteins, as TIA represents a mid-way point on the ACVS continuum.

## Materials and Methods

### Study Population

Patient enrollment took place over a 2-month period in 2015 during daytime hours at one Vancouver Island, Canada hospital. No a priori power analysis was done for this exploratory study. We restricted our study population to 40 patients and enrollment ceased immediately when we hit target; as this small study was serving the purpose of feasibility for a larger TIA biomarker study.

*Cases* constitute 20 stroke patients enrolled from the emergency department at one urban hospital who presented less than 24 h since symptom onset and who were managed under the hospital hot-stroke protocol. Patients with uncertain diagnosis, hemorrhagic stroke, and those unable to have medical imaging were excluded. Final diagnosis for cases and controls was made by a stroke neurologist and confirmed with diagnostic imaging. Diagnosis of stroke was made when patients had sudden onset of neurological deficit in a vascular distribution lasting > 24 h with restriction of diffusion on MRI, if used, or by CTA when intracranial occlusion corresponded with the side and pattern of symptoms. Adjudication for stroke etiology was done using modified TOAST classification [19].

*Controls* constitute 20 patients recruited concurrently from the stroke rapid assessment unit, which services ED and general practice (GP) referrals. The control patients were all-comers referred to the stroke unit and invited to participate with enrollment ceasing once the 20 patient target was met. Criteria for referral to the stroke unit is made at the discretion of the referring ED or GP physician and does not necessarily equate with a negative work up in the ED (or GP office). Cases were selected based on representation of a proportion of the population that would constitute stroke-mimics. The breakdown by referral source for the control patients was 65% (*n* = 13) ED and 35% (*n* = 7) general practice. The majority of controls were seen in the unit within 24 h of their ED or GP consultation with the highest range at 59 days.

### Study Procedures

Upon study enrollment, stroke nurses drew blood into 6-mL EDTA tubes using one of three different needle gauges depending on clinical need; 18 gauge butterfly with vacutainer (57.5%), 20 gauge (37.5%), or 21 gauge (5.0%). We have reported elsewhere on the slight impact of blood draw techniques (e.g., needle gauge) on proteomic levels [20]. Tubes were immediately iced until centrifuged for 10–15 min at 2500–3000 rpm at room temperature. Within 90 min of blood draw, 300 μL of plasma was pipetted into each of 32 (0.50 mL polypropylene) aliquots (3744, Thermo Scientific) per sample, then stored at − 80 °C.

### Proteomic Analyses

We used two MS techniques, direct and antibody enriched LC/MRM-MS (liquid chromatography multiple reaction monitoring mass spectrometry, henceforth MRM-MS), and commercial ELISA kits to measure the plasma levels of 147 proteins total, with some proteins being measured by multiple techniques (see “Results”). Direct MRM-MS was used to measure 141 proteins and the sample preparation protocol is similar to a previously reported procedure [21]. For low-abundance, endogenous proteins, we enriched from plasma using a mixture of eight antibodies (against EGFR, FABP, IL-6, PECAM, Prolactin, Protein S100-A12, Dickkopf-related protein 1, and Glutathione S-transferase P) coupled to Protein G-coated magnetic beads (Dynabeads® ThermoFisher Scientific) before proteolytic digestion with trypsin. MRM-MS data was processed with Skyline Daily 3.5.1.9426 analysis software [22]. ELISA was used for 32 proteins anticipated to be present at low abundance or where no suitable peptides were available for MRM-MS. See Online Resource for further details about plasma sample preparation, proteomic analyses, and ELISA kit information.

### Data Processing

Prior to statistical analysis, protein measurements below or above the lower and upper limit of quantitation (LOQ) were imputed to 0.5 and 1.5 times the smallest and largest observed values for that protein, respectively. The log2 transformed abundance (ELISA) and relative abundance (i.e., endogenous and SIS peptide ratios; MRM-MS) values were used as the protein response.

### Statistical Analysis

Statistical analyses were performed using R 3.3.1 [23]. Descriptive statistics were computed for the clinical variables and the protein measurements. Continuous clinical variables were compared between the two groups with *t* tests and categorical variables with Fisher’s exact test. The average protein levels were compared between the two groups, after adjusting for age, using a robust regression model (R package “robustbase” [24]) to reduce the impact of outliers. Proteins found to be differentially abundant between strokes and controls mimics with FDR < 0.20 were considered to be significant and retained for further evaluation as a putative biomarker panel. The effect of time from symptom onset on protein expression was examined within the 20 stroke patients using a linear regression model controlling for age and gender. The Benjamini-Hochberg procedure was used to compute FDR-corrected *p* values for the univariate analysis to address the issue of multiple comparisons [25].

To assess the potential value of a biomarker panel constructed from the significant proteins, we used principal component (PC) analysis (R package “pcaMethods” [26]) for dimension reduction and to summarize the information across all proteins [27]. The first two PCs were used to visualize the distribution of the proteins between controls and strokes. A multivariate logistic regression model was then constructed using PC1, PC2, and age; the fit of this model was then compared, with a likelihood ratio test, to a logistic regression model using only age. By controlling for age in this way, we seek to evaluate the diagnostic ability of the protein panel alone, removing the marked age effect for differentiating between the two groups.

Receiver operating characteristic (ROC) analyses were performed using the predictions from the logistic regression models and area under the curves (AUCs) were adjusted for optimism using two cross-validation (CV) methods: leave-one-out (LOO) and leave-pair out (LPO) (R package “pROC” [28]). CV estimates a classifier’s performance on an independent validation set [27]. LOOCV, which uses every case individually as a hold-out set, can produce biased estimates of the optimism-adjusted AUC in small data set; LPOCV is an alternative that generates unbiased optimism-corrected AUC estimates by using every pairwise combination of case and control as a hold-out set [29].

Protein interaction network analysis of the differentially abundant proteins was performed using STRING (version 10.0, string-db.org) [30], which integrates interaction data from several sources with information on physical and functional properties and with known and predicted protein interactions. In the protein interaction network, each protein is represented by a node and each interaction by an edge. This provides a better understanding of the biological pathways in which the most significant biomarkers were involved. For STRING analysis, the minimum required interaction score was set to high-confidence (0.7).

## Results

### Baseline Characteristics

Table 1 displays the demographic characteristics of the 20 cases (stroke) and 20 control patients, including medical history and concurrent medications for the stroke cases. The proportion of male and female patients did not differ between the two groups (*p* = 1.00); however, the stroke patients were older (*p* < 0.001). There were no missing data on the demographic and diagnostic categories for either group. In Table 2, we show that all patients had CT head investigations during their ED visit, and CTA was completed on 80% (*n* = 15) of strokes and of those 15 CTA, an MRI was also done on 2 of the stroke cases. All CT head and CTA investigations were conducted within 24 h of the patient presenting and in the majority of cases done within the immediate hours of the ED visit. MRI was done up to 2 h of the event window. In terms of concurrent medications, the stroke cases were relatively under-medicated with 40% (*n* = 8) of stroke patients on antiplatelet therapy for > 7 days, and less than 1/3 of the stroke cases on statins (*n* = 6; 30%). None of the stroke cases were on novel oral anticoagulants at the time of admission. Two of the 20 stroke cases received intravenous TPA as part of their hyper-acute patient management during the ED visit but the TPA administration was done *after* the blood collection for the study.

**Table 1:** Demographic summary for the case (stroke) and control patients

	Case (*n* = 20)	Control (*n* = 20)
Male	8 (40%)	7 (35%)
Age in years, median [range]	77 [46, 95]	63 [36, 77]
Previous medical history		
Atrial fibrillation	6 (30%)	0 (0%)
Diabetes	0 (0%)	1 (5%)
Hypertension	13 (65%)	7 (35%)
Hyperlipidemia	8 (40%)	7 (35%)
History of migraine without aura	0 (0%)	2 (10%)
History of migraine with aura	2 (10%)	1 (5%)
Concomitant medications at time of ED presentation		
Statin for at least the last 30 days	6 (30%)	4 (20%)
Antiplatelets for at least the last 7 days	8 (40%)	3 (15%)
Vitamin K antagonist	4 (20%)	0 (0%)
Novel anticoagulant	0 (0%)	0 (0%)
Smoking status		
Current smoker	5 (20%)	1 (5%)
Past smoker^a^	3 (15%)	9 (45%)

^a^Past smoker status was unavailable for one control and five stroke cases

**Table 2:** Diagnosis summary for the case (stroke) and control patients

	Case (*n* = 20)	Control (*n* = 20)
MRI positive	1 (5%)	0 (0%)
MRI negative	1 (5%)	1 (5%)
MRI not done	18 (90%)	19 (95%)
CTA abnormal	15 (75%)	0 (0%)
CTA normal	0 (0%)	12 (60%)
CTA not done	5 (25%)	8 (40%)
Localization		
Anterior circulation	19 (95%)	–
Left hemisphere	15 (75%)	–
Right hemisphere	4 (20%)	–
Not specified	1 (5%)	–
Posterior circulation	0 (0%)	–
Both	0 (0%)	–
Either circulation possible	1 (5%)	–
Case clinical sub-diagnosis		
Cardioembolism	9 (45%)	–
Cryptogenic	6 (30%)	–
Large artery atherosclerosis	3 (15%)	–
Antiphospholipid syndrome	1 (5%)	–
Incomplete evaluation	1 (5%)	–
Control diagnosis		
Migraine aura without headache	–	5 (25%)
Transient global amnesia	–	4 (20%)
Vestibulopathy	–	3 (15%)
Multiple sclerosis	–	2 (10%)
Neuropathy	–	2 (10%)
Syncope	–	2 (10%)
Psychogenic/anxiety/hyperventilation	–	1 (5%)
Other—mechanical musculoskeletal	–	1 (5%)

The mean (± standard error) time from symptom onset to blood draw was 10 ± 1.8 h for strokes and 281 ± 76.5 h for mimics. The mean time from blood draw to freeze was 35 ± 2.7 min for strokes and 29 ± 1.0 min for mimics.

### Biomarkers

Of the 147 protein targets, 115 were quantified by direct MRM-MS alone, 5 by ELISA alone, 19 by direct MRM-MS and ELISA, 1 by enriched MRM-MS and ELISA, and 7 by all three techniques. Each of the MRM-MS proteins was represented by a single peptide, with the exception of MMP-9 and thrombospondin-1, which were both measured by three peptides. In total, there were 147 proteins and 151 peptide sequences. From this list of 147 protein targets, 4 based on direct MRM-MS were removed prior to statistical computation: (a) creatine kinase-B type and MMP-9 (peptide LGLGADVAQVTGALR) were not detected in any of the MRM-MS samples; (b) myosin-11 was only detected in two samples; and (c) elastin had the same relative intensities across all samples. Of the proteins measured by multiple techniques, FABP was measured with the wrong peptide for MRM-MS and CD40 ligand was undetected in any of the samples using commercial ELISA kits, thus their measurements were not analyzed further. From the remaining protein targets, 114 met quality control criteria^1^ and were carried forward in the analysis.

**Fig. 1:**
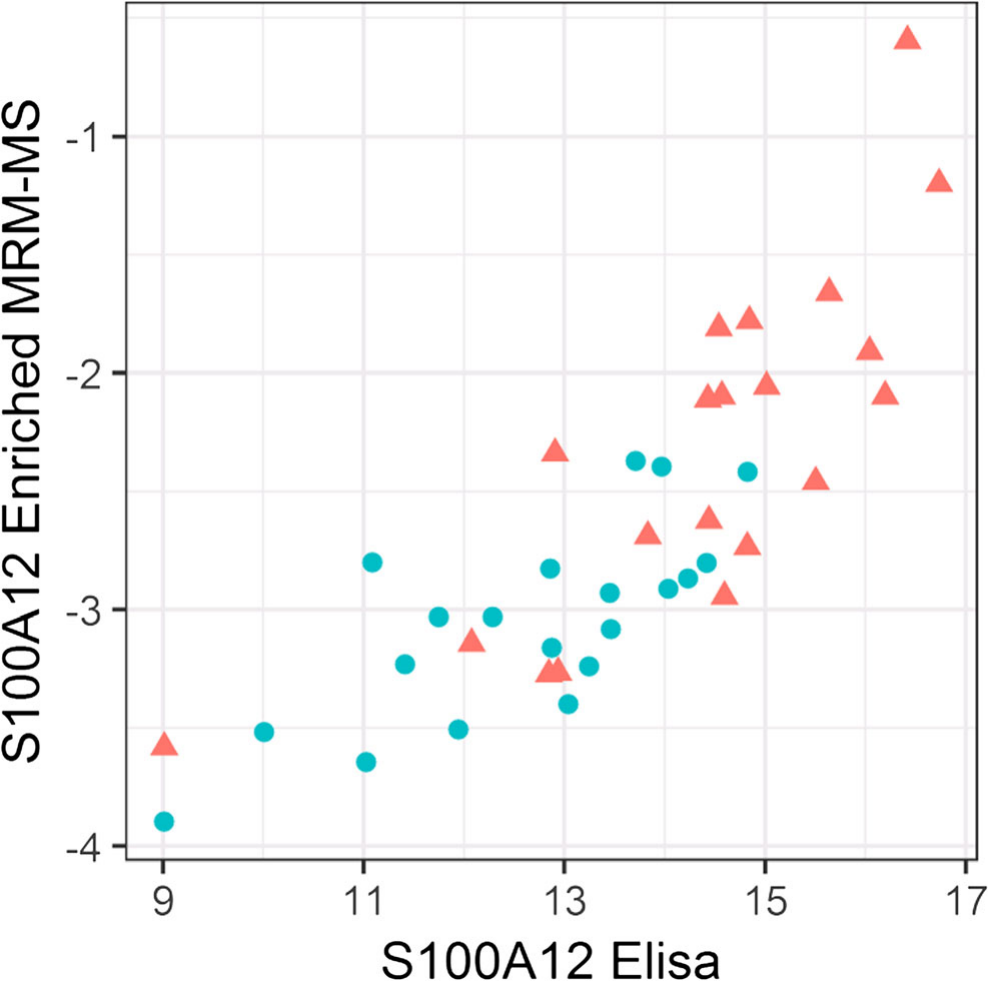
Scatterplot of enriched MRM-MS quantitation of S100A12 (log2 relative area) versus corresponding ELISA-based measurements (log2 abundance) for 20 strokes (triangle) and 20 controls (circle). The Pearson sample correlation is *r* = 0.82

Table 3 lists the 30 distinct proteins found to have differential abundances (FDR < 0.20) in the control and stroke patients after controlling for age in robust regression models. The significant proteins include 23 proteins based on MRM-MS, 4 based on ELISA, and 4 based on enriched MRM-MS. One of the differentially abundant proteins, S100A12, was measured by both enriched MRM-MS and ELISA thus providing an opportunity for comparison; the results for these two techniques were found to be well correlated (Pearson’s *r* = 0.82, see Fig. 1). In subsequent statistical analyses, we used the ELISA measurements of S100A12 rather than the enriched MRM-MS measurements. Among the 30 differentially abundant proteins, Prothrombin was highly correlated with Plasminogen (*r* = 0.83) and Vitamin K-dependent protein C (*r* = 0.90). All other protein pairs had correlations < 0.80. From the time-effect analysis for the 20 strokes where we controlled for age and gender in linear regression models, time did not have a significant effect on protein expression after FDR correction.

**Table 3:** Functional summary of the differentially abundant proteins (FDR < 0.20) identified by MRM-MS and ELISA

Protein name	UniProtKB ID	Protein symbol	Marker type and pathway map, if known	Control-mean (se)	Case (stroke) mean (se)	Age-adjusted robust regression *p* value	FDR-corrected *p* value
MRM-MS measured							
E-selectin	P16581	SELE	Infl	− 1.69 (0.03)	− 2.05 (0.05)	< 0.001	0.001
Apolipoprotein C-I	P02654	APOC1	Coag	1.23 (0.10)	0.32 (0.17)	< 0.001	0.003
Calponin	P51911	CNN1	AF	− 2.33 (0.06)	− 2.68 (0.07)	< 0.001	0.014
Coagulation factor XII	P00748	F12	Coag	0.17 (0.09)	− 0.37 (0.13)	< 0.001	0.014
Clusterin	P10909	CLU	Infl, complement pathways, CS	− 1.47 (0.05)	− 1.75 (0.05)	0.001	0.014
C-reactive protein	P02741	CRP	Infl, complement pathways	− 0.65 (1.38)	0.85 (0.38)	0.001	0.018
IGF-1	P05019	IGF1	Infl, cell adhesion, CS	− 3.54 (0.08)	− 4.16 (0.14)	0.001	0.018
Complement component 4b (C4b and C4a)	P0C0L5/ P0C0L4	C4B	Infl, complement pathways	0.03 (0.09)	− 0.32 (0.11)	0.002	0.029
Serum paraoxonase/ arylesterase 1 (Paraoxonase- PON1)	P27169	PON1	Infl	− 2.63 (0.08)	− 3.08 (0.09)	0.002	0.029
Prothrombin, thrombin	P00734	F2	Coag, platelet activation, CS	2.68 (0.05)	2.26 (0.08)	0.004	0.043
Plasminogen, plasmin, or angiostatin	P00747	PLG	Coag, platelet activation, cell adhesion	0.85 (0.06)	0.49 (0.07)	0.005	0.045
Vitamin K-dependent protein S (Protein S)	P07225	PROS1	Coag	− 1.42 (0.07)	− 1.59 (0.07)	0.010	0.078
Serum paraoxonase/ lactonase 3 (Paraoxonase- PON3)	Q15166	PON3	Infl	− 3.00 (0.11)	− 3.47 (0.09)	0.013	0.091
Vitamin K-dependent protein C (Protein C)	P04070	PROC	Coag	− 0.53 (0.07)	− 0.96 (0.09)	0.015	0.102
Antithrombin III	P01008	SERPINC1	Coag	− 0.53 (0.06)	− 0.84 (0.05)	0.018	0.114
Vitamin K-dependent protein Z (Protein Z)	P22891	PROZ	Coag	− 1.53 (0.13)	− 2.20 (0.21)	0.021	0.126
Coagulation factor V	P12259	F5	Coag, CS	− 3.75 (0.06)	− 3.94 (0.07)	0.022	0.126
Apolipoprotein D	P05090	APOD	Infl	− 0.72 (0.12)	− 0.92 (0.09)	0.026	0.137
Coagulation factor XI	P03951	F11	Coag	− 0.65 (0.08)	− 1.01 (0.10)	0.026	0.137
Insulin-like growth factor-binding protein 3 (IBP 3)	P17936	IGFBP3	Infl, CS	0.31 (0.07)	− 0.34 (0.15)	0.027	0.140
L-selectin	P14151	SELL	Infl	− 5.70 (0.07)	− 5.97 (0.06)	0.035	0.171
Plasma protease C1 inhibitor (C1 inhibitor)	P05155	SERPING1	Coag, complement pathways, cell adhesion	3.33 (0.07)	3.16 (0.06)	0.043	0.192
Plasma serine protease inhibitor (Protein C inhibitor)	P05154	SERPINA5	Coag, cell adhesion	− 6.95 (0.07)	− 7.49 (0.21)	0.044	0.192
ELISA measured							
Interleukin 6 (IL-6)	P05231	IL6	Infl, CS	− 0.77 (0.44)	2.67 (0.46)	0.002	0.029
S100A12	P80511	S100A12	Infl	12.63 (0.35)	14.37 (0.40)	0.002	0.029
Fatty acid binding protein 3 (FABP3)	P05413	FABP3	Neurovascular unit injury	3.00 (0.14)	4.31 (0.20)	0.008	0.075
Guanylate cyclase A (NPR1) (ANPR1)	P16066	NPR1	AF	− 1.60 (0.42)	− 0.16 (0.43)	0.046	0.192
Enriched MRM-MS measured							
S100A12	P80511		Infl	− 3.05 (0.09)	− 2.32 (0.17)	0.009	0.075
Epidermal growth factor receptor (EGFR)	P00533		Infl	− 1.05 (0.06)	− 1.37 (0.06)	0.010	0.078
Platelet endothelial cell adhesion molecule (PECAM 1)	P16284		Infl	− 9.23 (0.42)	− 8.44 (0.36)	0.044	0.192
Prolactin	P01236		Hormone	− 3.05 (0.20)	− 2.18 (0.25)	0.045	0.192

Protein symbol reflects the terminology presented in Fig. 4. Marker type: Coag = coagulation, AF = atrial fibrillation, Infl = inflammation, CS = cancer signaling. Reported protein measurements are log2 abundance (ELISA) and relative abundance (MRM-MS) values

**Fig. 2:**
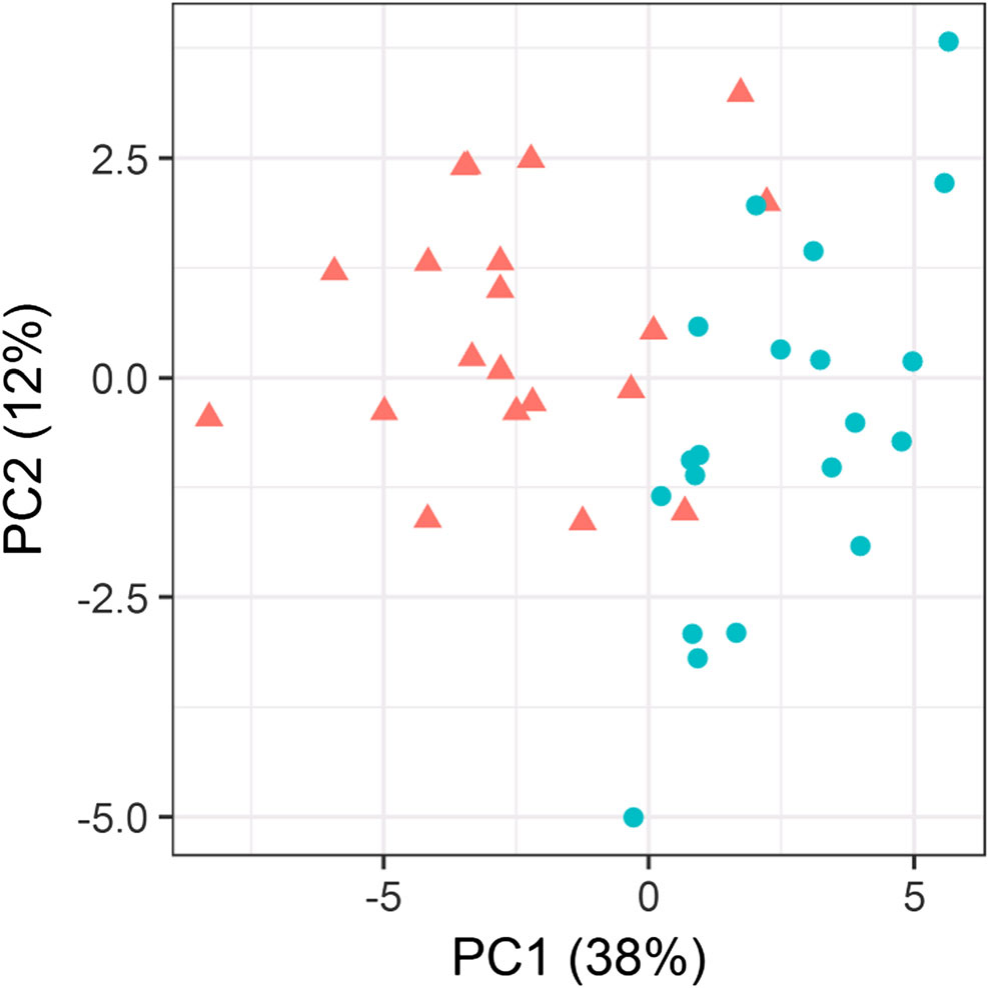
The first two principal components, PC1, PC2, of the 30 differentially abundant proteins clearly separate the strokes (triangle) and controls (circle)

**Fig. 3:**
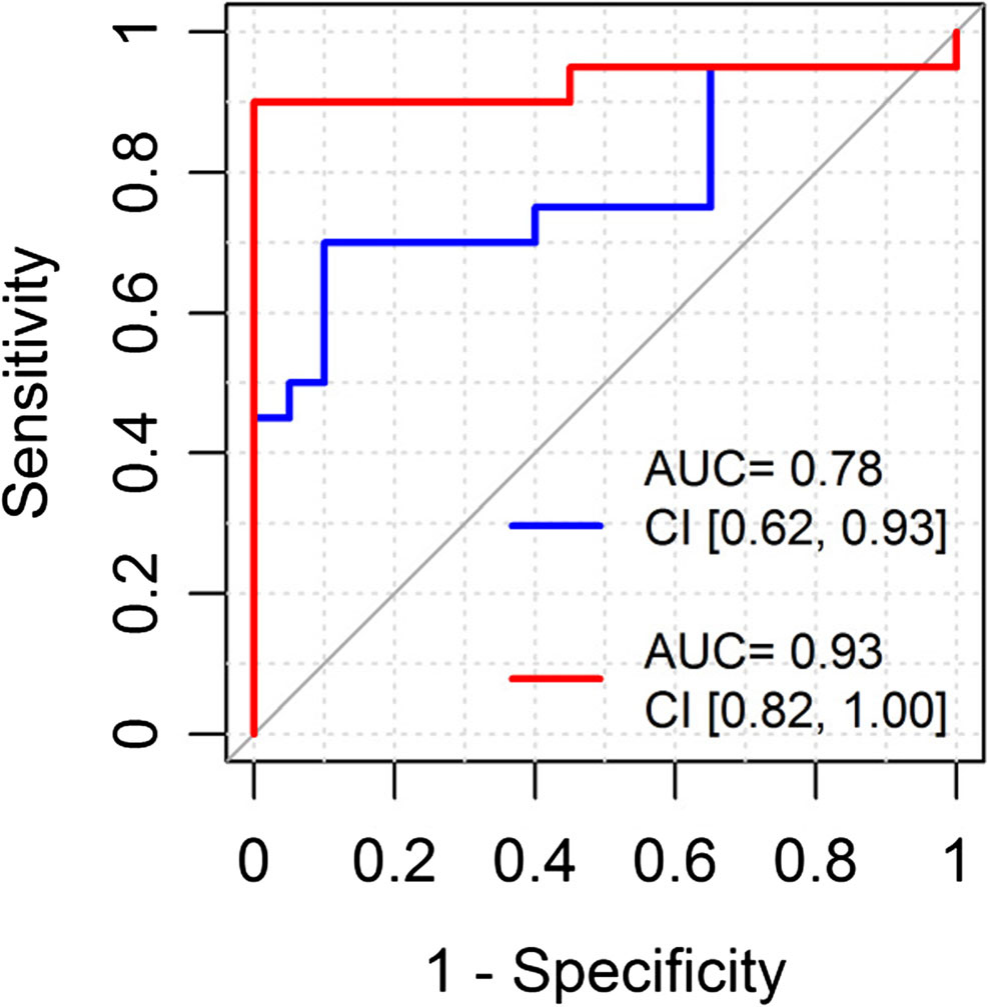
Receiver operating characteristic (ROC) plot adjusted by leave-one-out cross-validation comparing logistic classifiers based on age alone (blue) and age plus the first two principal components of the differentially expressed proteins (red). The 95% confidence interval (CI) for AUC is shown

**Fig. 4:**
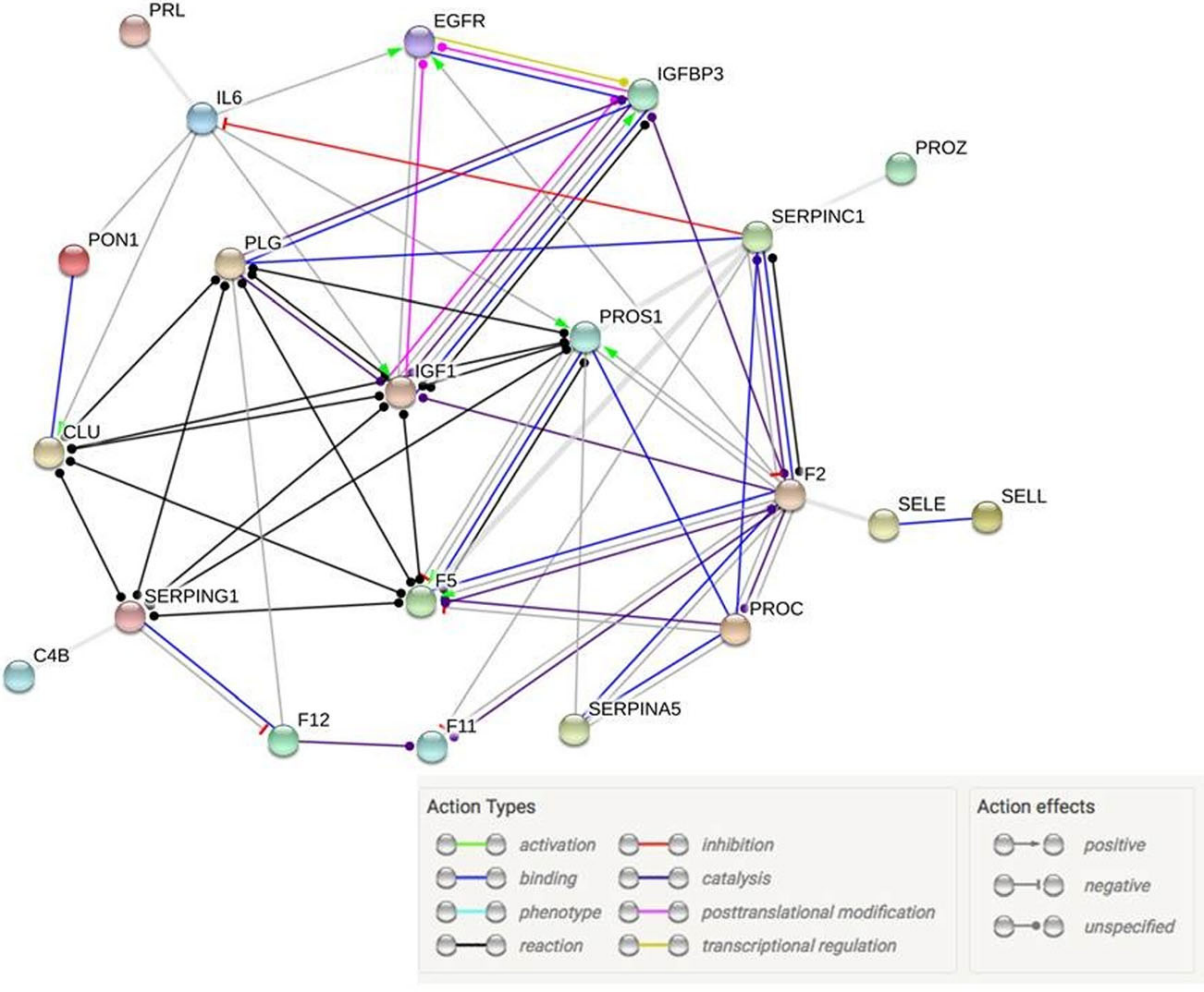
Functional interaction network of differentially abundant proteins visualized using STRING; interactions are coded by color and effects. See Table 3 for protein symbol reference

In a principal component analysis using these 30 differentially expressed proteins, the first two PCs explained 38% (PC1) and 12% (PC2) of the total variability (see Fig. 2). A logistic classifier incorporating PC1 and PC2 plus age showed significantly improved performance for separating the stroke and control patients compared to a model based on age alone (likelihood ratio test *p* < 0.001; LOOCV AUC (95% CI): 0.93 (0.82–1.00) vs. 0.78 (0.62–0.93); LPOCV AUC: 0.93 (0.90–0.96) vs. 0.80 (0.76–0.84); see Fig. 3 for the LOOCV AUC. The similar optimism-adjusted AUCs from LPO compared to LOO demonstrate the stability of our classifier. From the list of 30 differentially abundant proteins, STRING analysis showed 20 to have high-confidence molecular interactions with other proteins in this list (see Fig. 4). The remaining 10 proteins either did not interact or had interactions of low-confidence.

## Discussion

The management of ACVS could be greatly improved by the availability of robust, accessible, and inexpensive biomarkers. Clinical decisions that could benefit from a proteomic blood test include differentiating TIA from its many mimics, and in the future, a validated and clinically useful blood test could provide guidance to select candidates for thrombolysis and thrombectomy. A pre-requisite to candidate selection would be that the assay development and validation would necessarily be contingent upon plasma collected during a tight time windom from symptom onset that mirrors that of the hyper-acute context in which those interventions are delivered. To date, no individual protein biomarker has emerged for such critical decision support, but there are many interesting candidates. The answer may lie in finding patterns of biomarkers rather than single entities. Given that the majority of publications report on a single protein or a small group of proteins, typically two to five, this puts weight on the ability to gather sufficient data to find such patterns. This study demonstrates how one technique, LC/MRM-MS, can be used to achieve larger data sets, laying a foundation for further research to demonstrate the utility of MS in translational settings at the bedside with benchtop MS machines in laboratories or as point of care devices, for example.

We examined 147 high interest proteins and found a subset of 30 proteins that, in conjunction with age, could distinguish stroke from stroke-controls with a high AUC, thus achieving our goal of demonstrating the potential value of biomarker panel for ACVS diagnosis. The 30 significant proteins are involved in blood coagulation, inflammation, neurovascular unit injury, cell adhesion, and atrial fibrillation. Using STRING analysis, we identified 20 of them to have high-confidence molecular interactions with one another. The significant proteins and pathways highlighted here are consistent with the known biology of how proteins are up- or down-regulated during ischemic stroke.

The results of this study are limited by sample size. We acknowledge that 40 patients do not justify representation of the tremendous heterogeneity inherent in stroke and mimic populations, yet this was designed specifically as a pilot study to assess (i) feasibility of plasma collection, processing, consent, and logistics for the larger SpecTRA project (for TIA biomarkers) with enrollment that will surpass 1600 ED patients and (ii) detection of a proteomics signal in stroke to serve as a baseline against which we will compare our larger TIA biomarker study and its control group.

Further, our results are also limited by an imbalance in baseline patient characteristics between strokes and controls as we were not able to match patients in both groups for health and sociodemographic variables, such as age. We acknowledge that the generalizability of the results is affected by limiting the model to age. It is likely that measures of stroke severity and related comorbidities would be associated with the diagnosis of stroke. Patient age, though, is a clinical variable easily collected in patient care settings. Other clinical measures, such as time from symptom onset, are often imprecisely measured or reported by patients. However, the aim of our study was to assess whether MS platforms are capable of yielding useful information for the diagnosis of stroke. The results of our study suggest that after accounting for patient age, such is the case. The generalizability of these results may be attenuated by other competing clinical measures not included in our model (e.g., stroke size) that may share information with the proteins we assessed. In the larger TIA biomarker study, we demonstrate the discriminative capacity of these proteomic markers over and above readily available clinical variables that are often collected in the prehospital and acute care settings during stroke assessments, for example, vascular comorbidities, presence of atrial fibrillation, and motor deficit.

The generalizability of our results, though, we suggest, is a secondary consideration to our main aim of demonstrating that the MS platform in conjunction with a readily available clinical variable (i.e., age) could yield clinically useful information. Yet as we state above, detecting patterns of biomarkers may ultimately lead to greater clinical value than identifying single biomarker candidates. As such, our study is focused on demonstrating the diagnostic potential of the combination of protein biomarkers, as assessed by MS, rather than on the generalizability of any specific protein biomarker. Related to that, we note that with a MS approach there are challenges in measuring low-abundance proteins. An illustrative example is MMP-9 which figures highly in many previous studies but lies below the measurable threshold of regular LC/MRM-MS. Other limitations include the absence of time since onset in the analysis; this shortcoming has been addressed in our larger TIA study in which patients are enrolled based on symptom onset within 24 h. Finally, we did not include intracerebral hemorrhage at this stage of the project.

Despite this pilot study’s limitations, discriminative power was high, making the pursuit of further plasma biomarker validation studies using MS worthwhile. Further, the results add weight to the argument for multi-protein panels and the use of how one technique, LC/MRM-MS, can be used to achieve larger data sets, laying a foundation for further research to demonstrate the utility of MS in translational settings at the bedside with benchtop MS machines in laboratories or as point of care devices, for example. However, despite its capacity for highly multiplexed and very specific determination of protein abundance, it is clear that biomarker assays will necessarily require translation from the LC/MRM-MS research platform onto much more rapid and clinic-ready platforms (e.g., iMALDI, ELISA). Sample turnaround times measured in hours and days from blood draw to test result are not appropriate in the acute care setting; turnaround times measured in minutes would be required. Assays with these characteristics are the focus of several ongoing follow-up projects in this area.

Where we measured proteins by multiple techniques, only 1 of 27 was found to be differentially expressed at the 5% significance level by more than one method. It is not clear which technique gives the more clinically relevant results. This highlights the question of analytical validity that needs to be considered when we apply these various proteomic techniques in clinical medicine. Where ELISA may suffer from lack of specificity being confounded by the presence of different isomers or other proteins sharing epitopes, it can be very sensitive; on the other hand, MS is highly specific, but may lose sensitivity due to protein modification during processing or the presence of a variant protein in an individual. It is recognized that some plasma protein levels fluctuate significantly in the general population due to heritable factors, individual and common environmental factors, and as yet unknown factors [31]. Many potential stroke protein markers are low-abundance proteins (i.e., not easily or readily quantifiable) [7], and their variability in the general population is not well known.

These limitations notwithstanding, we are confident that the findings from this preliminary study are a useful contribution to the published literature, providing more contexts for those of us trying to navigate in the world of protein markers in stroke. Our aim was to explore variability of protein markers within these two small patient groups, recognizing diversity within disease and across healthy populations and not to *discover* stroke markers.

## Conclusion

A clinical reliance on concurrent multiplexed assays of large numbers of proteins is economically feasible using MS, and variants of this technology have the potential for use in clinical settings where stroke treatment decisions are made. Understanding a metabolic interrelationship of most of the 30 proteins we found differentially abundant between stroke and other patients who were referred for neurological consultation strengthens the notion that these are biologically significant associations rather than purely statistical correlations. The appeal of a proteomic descriptor of the ischemic process remains exciting, particularly as further studies add temporal, etiological, and prognostic detail to basic underlying patterns. This study is small and requires validation, but the strength of the observed protein signal holds promise.

## Footnotes

1 As a quality control measure, we deemed any protein with all log2 abundance levels less than − 8 to be essentially undetectable.
